# Five cases of paroxysmal kinesigenic dyskinesia by genetic diagnosis

**DOI:** 10.3892/etm.2014.2155

**Published:** 2014-12-22

**Authors:** GUO-HONG CHEN

**Affiliations:** Department of Neurology, Zhengzhou Children’s Hospital, Zhengzhou, Henan 450053, P.R. China

**Keywords:** paroxysmal kinesigenic dyskinesia, PRRT2, incomplete penetrance

## Abstract

Paroxysmal kinesigenic dyskinesia (PKD) is an autosomal dominant disorder and PRRT2 is the causative gene of PKD. The aim of this study was to investigate PRRT2 mutations in patients who were clinically diagnosed with PKD. Nine PKD cases, including four familial cases and five sporadic cases, were selected. Peripheral blood was drawn after obtaining informed consent, and genomic DNA was extracted by a standard protocol. Sanger sequencing was performed for the screening of PRRT2 mutations. A total of five cases were detected to harbor PRRT2 mutations. Four familial cases carried a c.649dupC (p.Arg217Profs^*^8) mutation, while one sporadic case and his asymptomatic father carried a c.133-136delCCAG (p.Pro45Argfs^*^44) mutation. PRRT2 mutations were not identified in the remaining cases. The study further confirmed that PRRT2 was a causative gene of PKD and implied that PRRT2 mutation has incomplete penetrance.

## Introduction

Paroxysmal kinesigenic dyskinesia (PKD), also known as paroxysmal exercise-induced dyskinesia syndrome, is an autosomal dominant genetic disease, the incidence rate of which is ~1/150,000 (1). The clinical features are sudden exercise-induced dance-like movements of the limbs or torso, throwing disorders, stiffness or numbness, dystonia and other actions (2). PKD is mostly onset in childhood and increases in prepubescence; however, the symptoms are spontaneously relieved in adulthood and disappear at an age of 30–40 years (3). The patients have clear consciousness at onset, and exhibit normal electroencephalograms during seizures and the interictal period, which differentiates it from epilepsy. Patients with PKD are very sensitive to antiepileptic drugs; small doses of carbamazepine or phenytoin can effectively control the seizures of PKD, and the symptoms may disappear completely in certain patients after treatment with drugs for three days (4).

Since it was first reported in 1967 (5), it has been confirmed that there are two pathogenic gene regions in PKD, specifically 16p11.2-q12.1 and 16q13-q22.1 (6,7). There may be a third pathogenic region in PKD; however, this is not on chromosome 16 (8). Nevertheless, the causative gene has not been cloned. In 2011, Chen *et al* collected eight families with PKD and discovered using whole exon sequencing combined with Sanger sequencing that all eight families were carrying PRRT2 gene mutations, while no PRRT2 mutations were found in the 1,000 cases of the normal control group (9). Therefore, Chen *et al* identified PRRT2 as a causative gene of PKD, which was confirmed in subsequent studies (10–12). However, there is little literature concerning PKD, and genetically confirmed cases of PKD are rarely reported. In the present study, nine cases of clinically diagnosed PKD were collected from 2007 onwards, and the genes of these patients were sequenced by the Sanger method. The incidence of PRRT2 mutations in the patients was determined to investigate PRRT2 as a pathogenic gene in PKD.

## Materials and methods

### Subjects

The study included nine patients with PKD (cases PKD 1–9) from seven different pedigrees (families 1–7). These included six male cases (PKD 2, 3, 5 and 7–9) and three female cases (PKD 1, 4 and 6). Four patients had a family history (PKD 1–4) and the other five were sporadic cases. Family 1 had eight members, three of whom had a history of PKD, but the patient 1 refused a blood sample and thus it was not possible to determine whether that patient carried PRRT2 mutations. The family 2 proband exhibited involuntary twisting of the body and his father mainly complained of sudden movement. The remaining five probands and their parents were asymptomatic and classified as sporadic cases. The present study was approved by the Ethics Committee of Zhengzhou Children’s Hospital (Zhengzhou, China). All patients provided informed consent.

### Blood sample collection

Venous blood samples (3 ml) were obtained for anticoagulation by sodium citrate. They were stored in a refrigerator at 4°C for short periods, and frozen at −20°C for long-term preservation.

### DNA extraction

The QIAamp blood DNA extraction kit (Qiagen, Hilden, Germany) was used to extract DNA from the blood samples. The DNA was dissolved with 1X AE solution (Qiagen), and stored at −20°C for the long-term.

### Polymerase chain reaction (PCR)

Five PRRT2 primers were used according to the literature (9), and are shown in (Table I). Four exons including exon-intron joints (≥20 bp) were amplified. The PCR annealing temperature was 66°C (exon 1) or 60°C (exon 2a, 2b, 2c, 3–4).

### Sequencing

To 5 μl PCR product was added 0.3 μl shrimp alkaline/heat-sensitive alkaline phosphatase [Promega (Beijing) Biotech Co., Ltd., Beijing, China], 0.2 μl exonuclease [New England Biotechnology (Beijing) Co., Ltd., Beijing, China] and 2.5 μl ddH_2_O for purification. The mixture was placed in a 37°C water bath for 1 h, then in a 80°C water bath for 15 min. The 3 μl purified PCR product that was obtained was added to 1 μl 3.2 pmol/l unidirectional primer and 1 μl BigDye working fluid (Beijing Yue Wei Gene Technology Co., Ltd., Beijing, China) for forward sequencing reaction. The reaction conditions were: 96°C denaturation for 1 min; 94°C denaturation for 10 sec, 50°C annealing for 5 sec and 60°C extension for 4 min, for 25 cycles; followed by 60°C extension for 10 min. Following the sequencing reaction, 50 μl 75% ethanol mixture was added and the resultant mixture was stored at −20°C in a refrigerator for 60 min. After centrifuging at 10,800 × g for 30 min, the supernatant was discarded, 50 μl 75% ethanol was added and further centrifugation at 10,800 × g for 30 min was conducted. The supernatant was discarded. After drying, 7 μl Hi-Di (Shanghai Top Zhuo Biotechnology Co., Ltd., Shanghai, China) was added to the residue, which was the loaded onto an ABI 3730 genetic Analyzer (Haimai Pu Biotechnology Co., Ltd.) for sequencing.

## Results

### Clinical manifestations

**Figure 1:**
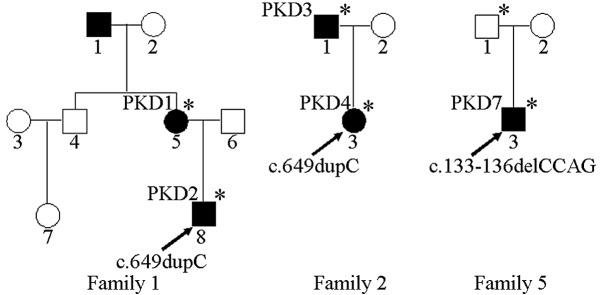
Pedigree chart for the patients with paroxysmal kinesigenic dyskinesia (PKD). − indicates male, ○ represents female, □ or ● denotes affected individuals, arrow indicates proband, ^*^indicates positive for PRRT2 mutation.

The clinical manifestations of the nine patients with PKD are shown in Table II, and the familial pedigrees of five of the cases are shown in Fig. 1. The proband (PKD 2) in family 1 had the chief complaint of involuntary limb swinging when starting to run that was automatically relieved after lasting for ~30 sec. The consciousness of the patient was clear at onset and the seizures occurred 3–10 times per day. The proband’s mother (PKD 1) reported dance-like movements of the upper limbs at the age of 10 years; however, the symptoms were mild and improved without treatment. Questioning revealed that the proband’s grandfather had a history of dance-like movements of the upper limbs. The proband (PKD 4) in family 2 involuntarily stood from a seated position with a twisted body posture and strange facial expressions, and the father (PKD 3) had similar attacks at the ages of 10–18 years but no longer experienced symptoms. Sporadic case PKD 5 had double-leg spasms in sudden movements, each episode lasting for ~10 sec. PKD 6 and PKD 9 had lower limb stiffness in a sudden movement or tensing, which was sustained for 5–15 sec; PKD 9 sometimes fell down. PKD 7 experienced involuntary limb swinging in sudden movements. PKD 8 had left upper limb spasticity in sudden movements or when changing position.

### PRRT2 gene sequencing

**Figure 2:**
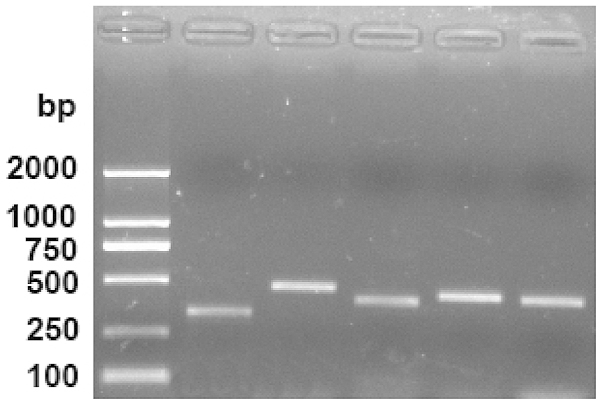
PCR gel results of four PRRT2 gene exons. M, DNA marker; lanes 1–5: exon 1, exon 2a, exon 2b, exon 2c and exons 3–4, respectively.

A gel map following PCR amplification is shown in Fig. 2. The amplification bands of the five PCR primers were 329, 467, 398, 433 and 431 bp respectively.

**Figure 3:**
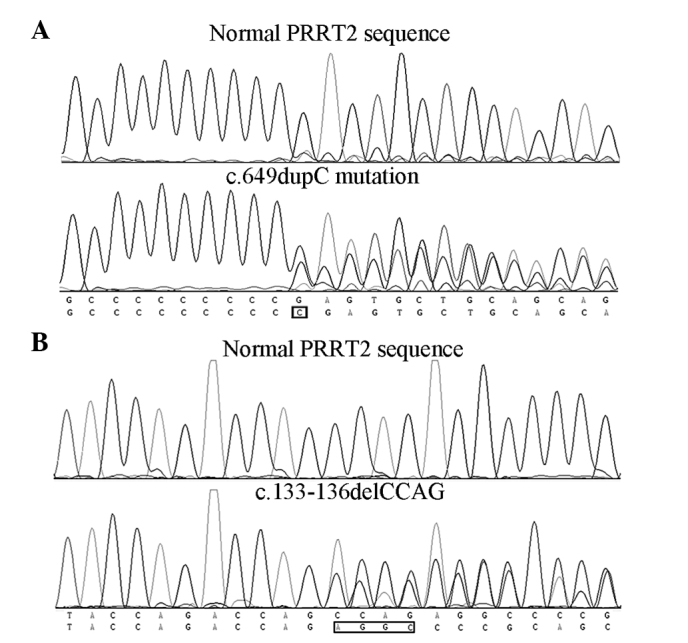
Normal PRRT2 and (A) c.649dupC and (B) c.133-136delCCAG mutated gene sequences.

Sanger sequencing results showed that PKD 1–4 carried the PRRT2 gene c.649dupC (p.Arg217Profs*8) mutation (Fig. 3A) and PKD 7 carried the c.133-136delCCAG (p.Pro45Argfs*44) mutation (Fig. 3B), while no PRRT2 gene mutations were identified in the remaining patients. Since PKD 7 was a sporadic case, further screening for the mutation was performed and it was found that the asymptomatic father also carried the mutation.

## Discussion

PKD is the most common form of paroxysmal dyskinesia with onset triggered by sudden movements. Since PKD was first reported in 1967, a number of academics and clinicians have been committed to identifying the genes that cause PKD. In 2011, Chen *et al* reported internationally that PRRT2 was a PKD disease-associated gene (9), and this was soon confirmed by other research groups (10–12). It has now been confirmed that PRRT2 is a causative gene of PKD. The findings of Chen *et al* provided the foundation for the clinical molecular diagnosis of PKD.

However, few studies have reported on PKD. Song and Hu described in detail the clinical manifestations of five PKD cases (13); Li *et al* reported seven PKD patients (14) and Lin *et al* reported three PKD pedigrees with clinical and genetic characteristics (15). Mao *et al* reported the clinical features of 34 PKD patients (16). However, PKD cases confirmed by genetic methods were not reported. In the present study, nine cases with a clinical diagnosis of PKD were collected, including four cases with a family history. It was found that all four cases with familial PKD and one case with sporadic PKD carried PRRT2 mutations by Sanger sequencing of the PRRT2 gene, which further confirmed that PRRT2 is a causative gene of PKD. In family 5, the proband PKD 7 carried a PRRT2 gene mutation at the c.133-136delCCAG site; however, further testing found that the asymptomatic father of PKD 7 also carried the same genetic mutation. This suggests that PRRT2 mutations have incomplete penetrance, that is, that certain individuals carrying the gene mutations of PKD do not exhibit clinical symptoms. The reason for the incomplete penetrance of PRRT2 gene mutations may be influenced by the environment or other genetic modifications.

Currently, >30 PRRT2 mutations have been identified in patients with PKD (9–12,17–21), of which >95% are truncated mutants, while only ~5% are missense mutations and splicing mutations (22). In the PRRT2 gene mutations, c.649dupC is the most common site, accounting for ~80% of cases (19). The high incidence of the mutations has prompted c.649dupC to be defined as a high-frequency mutation. In the present study, four of the five patients carrying PRRT2 mutations had c.649dupC mutations, accounting for 80%, which confirms the high frequency of this mutation. The study by Li *et al* found that c.649dupC mutations can be *de novo* mutations (23), suggesting that the mutation was not from a founder effect but was a hotspot mutation. Other studies support that c.649dupC is a hotspot mutation (18,19).

Although PRRT2 genes of PKD have been cloned, the function of PRRT2 remains unclear. Co-immunoprecipitation results suggest that PRRT2 interacts with SNAP25 (12,24). SNAP25 is a presynaptic membrane protein involved in the docking and fusion of synaptic vesicles and associated with the release of neurotransmitters (25). Since SNAP25 forms a SNARE complex with syntaxin and synaptobrevin (26), this suggests that PRRT2 may have functional connectivity with the SNARE complex, or may be a component of the SNARE complex. In addition, SNAP25 induces synaptic vesicles to release neurotransmitter when triggered by Ca^2+^, indicating that PRRT2 mutations are likely to cause abnormalities in neurotransmitter release (27). Whether PRRT2 mutations affect intracellular Ca^2+^ influx remains unclear, and requires further investigation.

The present study confirmed that PRRT2 is a pathogenic gene of PKD, and that c.649dupC is a high frequency mutation site. In addition, the presence of incomplete penetrance of PRRT2 was revealed.

## Figures and Tables

**Table I:** PRRT2 gene forward and reverse primers.

Exons	Forward primers (5′→3′)	Reverse primers (5′→3′)
Exons 1	TTGCCTGGGTAACGCGTGGCT	ACACCCGCATTCCCGTGCAGT
Exons 2a	CAATT GGGCCTGCAGTGCTGAG	GGTTTGGACACTGTTTCTTGGCAT
Exons 2b	GGAGGGGAATCAAAGGCCAACTG	TCAACCAGCTGCTGCAGCACTC
Exons 2c	GAAAAGCAAGAGAATGGGGCAGTG	GATTACTCCAGAGGCTCTATTGCAG
Exons 3–4	TTCTGGATGACTTTTCCACCTGAT	CAACAGGAAGAAAAGTCTTGGGAT

**Table II:** Clinical data of PRRT2 gene test results for nine patients with paroxysmal kinesigenic dyskinesia (PKD).

Number	Gender	Family history	Onset age (years old)	Main symptoms	Seizure frequency	Drug treatment	PRRT2 mutation
PKD 1	Female	Familial	10	Upper limb dance-like movements	No symptoms currently	Untreated	c.649dupC
PKD 2	Male	Familial	9	Involuntary limb swinging	3–10 times per day	Carbamazepine	c.649dupC
PKD 3	Male	Familial	10	Involuntary twisting of the body	No symptoms currently	Phenytoin sodium	c.649dupC
PKD 4	Female	Familial	8	Involuntary twisting of the body	1–5 times per day	Carbamazepine	c.649dupC
PKD 5	Male	Sporadic	11	Lower limb spasms	1 time per 2–3 days	Carbamazepine	None
PKD 6	Female	Sporadic	12	Lower limb stiffness	1–3 times per day	Sodium valproate	None
PKD 7	Male	Sporadic	5	Involuntary limb swinging	3–5 times per day	Carbamazepine	c.133-136delCCAG
PKD 8	Male	Sporadic	10	Left upper limb spasm	5–20 times per day	Carbamazepine	None
PKD 9	Male	Sporadic	12	Lower limb stiffness and falling down	1–5 times per day	Oxcarbazepine	None
